# The Impact of Artificial Intelligence on Firm Performance: An Application of the Resource-Based View to e-Commerce Firms

**DOI:** 10.3389/fpsyg.2022.884830

**Published:** 2022-04-07

**Authors:** Donghua Chen, José Paulo Esperança, Shaofeng Wang

**Affiliations:** ^1^School of Logistics and e-Commerce, Zhejiang Wanli University, Ningbo, China; ^2^ISCTE Business School, BRU-IUL, University Institute of Lisbon, Lisbon, Portugal; ^3^Smart Learning Institute, Beijing Normal University, Beijing, China

**Keywords:** artificial intelligence capability, firm performance, resource-based view, PLS-SEM, firm creativity, driven decision making, environmental dynamism, innovative culture

## Abstract

The application of artificial intelligence (AI) technology has evolved into an influential endeavor to improve firm performance, but little research considers the relationship among artificial intelligence capability (AIC), management (AIM), driven decision making (AIDDM), and firm performance. Based on the resource-based view (RBV) and existing findings, this paper constructs a higher-order model of AIC and suggests a research model of e-commerce firm AIC and firm performance. We collected 394 valid questionnaires and conducted data analysis using partial least squares structural equation modeling (PLS-SEM). As a second-order variable, AIC was formed by three first-order variables: basic, proclivity, and skills. AIC indirectly affects firm performance through creativity, AIM, and AI-driven decision making. Firm creativity, AIM, and AIDDM are essential variables between AIC and firm performance. Innovation culture (IC) positive moderates the relationship between firm creativity and AIDDM as well as the relationship between AIDDM and firm performance. Environmental dynamism (ED) positive mediates the connection between AIM and AIDDM. Among the control variables, firm age negatively affects firm performance, and employee size does not. This study helps enterprises leverage AI to improve firm performance, achieve a competitive advantage, and contribute to theory and management practice.

## Introduction

The rapid evolution of artificial intelligence (AI) brings enterprises more business opportunities (Hughes et al., 2020; Obschonka and Audretsch, 2020; Shareef et al., 2021). Artificial intelligence is the machines (programs) that operates in the simulation of human intelligence (Łapińska et al., 2021) in technologies, such as machine learning, data mining, natural language processing, image recognition, etc. (Khalid, 2020). Artificial intelligence can bring efficiency gains, cost savings, product quality improvements, and customer service improvements (Bag et al., 2021c). Enterprise capabilities are critical for identifying business opportunities (Yao et al., 2021). While there is excellent potential for artificial intelligence capability (AIC) to improve a company’s performance (Mikalef and Gupta, 2021), there are also significant challenges to these companies applying AI (Yu et al., 2021). Businesses can utilize AI to improve the customer service experience by offering more appropriate recommendations and less costly options (Payne et al., 2021). According to the resource-based view (RBV; Majhi et al., 2021); artificial intelligence’s applied capability is an ensemble of implicit resources (Bag et al., 2021c). These resources include supporting resources, labor skills, and organizational coordination (Kim, 2019; Selz, 2020). Once a firm masters organizing resources that are impossible to copy effortlessly, it possesses a competitive advantage (Yasmin et al., 2020) and enhances firm performance (Chen and Lin, 2021). Therefore, there is an essential theoretical and practical value in exploring the mechanisms and critical factors of the impact of AIC on firm performance (Chen and Lin, 2021; Mikalef et al., 2021), especially in the e-commerce industry with direct customer contact (Wang and Fan, 2021).

A broad study of the impact of AI and its capability on business performance appears (Denicolai et al., 2021; Mikalef and Gupta, 2021). The existing literature dedicated to the study of the impact of AI on industries, such as banking and finance (Huynh et al., 2020), manufacturing (Bag et al., 2021c), automated retailing (Pillai et al., 2020), logistics (Chien et al., 2020), marketing (Keegan et al., 2022), coaching services (Kim et al., 2021b), and customer relationship management (Chatterjee et al., 2021a), among other areas. In comparison, these studies concentrated on the impact of AI on firm innovation processes and management practices, technological innovation (Liu et al., 2020a), and the relationship between AI learning and entrepreneurial performance (Khalid, 2020). In e-commerce, technology applications of AI are also proliferating nowadays (Volkova et al., 2021). For example, e-commerce firms predict the most acceptable promotion targets (Giannoulakis, 2020) and pricing strategies (Shang et al., 2020) founded on consumers’ recorded user profiles, trajectories, and consumption history. E-commerce firms’ consumer product recommendations are built on robust data analysis (Li et al., 2021). The AI customer service can help customers solve problems quickly (Varsha et al., 2021). E-commerce companies can deepen exploration and analysis under past data to capture market trends to improve operational efficiency (Cui et al., 2021). However, little is known about the mechanism of AIC composition of e-businesses and AIC’s impact on e-business performance (research gap 1).

Enterprise creativity is a key to generating new ideas, products, and services (Yao et al., 2021) and is a potential factor simulating business performance (Mikalef and Gupta, 2021). Big data can enhance AIC (Ghasemaghaei, 2021) and decision-making for more profitable business outcomes (Denicolai et al., 2021). The AI-related business management systems are essential factors in optimizing business performance (Rahman et al., 2021). Despite the great potential of AI technologies to facilitate firm performance, the current publications rarely focus on the firm creativity, artificial intelligence management (AIM), and artificial intelligence-driven decision making (AIDDM) in the relationship between the two (research gap 2). Dynamic changes in the business environment may influence the application of digital technologies represented by AI (Bag et al., 2020b) and firm performance (Dubey et al., 2020). Organizational culture of innovation may also be an essential variable affecting firm performance (Dubey et al., 2020). However, existing investigation have few moderating variables under innovation culture (IC) and environmental dynamism (ED) to demonstrate the relationship between AIC and firm performance (research gap 3).

To fill the current research gaps, this study will investigate the internal components of AIC. We will analyze the impact of AIC, management, driving decision making, firm creativity, innovation culture, and environmental dynamism on firm performance, using e-commerce firm performance. We constructed the higher-order variable of AIC, the theoretical model of the impact of AIC on e-commerce firm performance, and the corresponding research hypotheses relating to the RBV and existing relevant research results. We use partial least squares structural equation modeling (PLS-SEM) analysis to empirically analyze 394 valid questionnaires to test further hypotheses and theoretical models proposed in this study. Based on the data analysis, we discussed the effects of higher-order variables (AI capability), moderating variables (innovation culture and environmental dynamism), and mediating variables (firm creativity, AIM, and AIDDM) on firm performance. This study makes theoretical contributions to AI and firm performance and provides essential guidance for e-commerce companies to improve their performance and develop a competitive advantage.

## Literature Review and Conceptual Model

### Resource-Based View

The RBV believes that essential resources determine firm performance (Barney, 1991; Chatterjee et al., 2021b). Resources can be tangible and intangible assets within an organization (Mikalef and Gupta, 2021). According to this theory, valuable, rare, inimitable, and irreplaceable resources can build a competitive advantage by creating value and improving firm performance (Barney, 1991; Ghasemaghaei, 2021). Such an advantage can persist over a long period (Bag et al., 2021c). Businesses can raise the value of their resources because the combined value of the complementary resources is higher than the sum of each resource (Ghasemaghaei, 2021; Mikalef et al., 2021).

Artificial intelligence capability is increasingly a critical and intangible resource for business performance advancement (Belhadi et al., 2021; Lou and Wu, 2021; Mikalef and Gupta, 2021). It suggests that artificial intelligence may bring a competitive advantage to businesses (Chaudhuri et al., 2021). AIC can deliver businesses access to valuable, rare, inimitable, and irreplaceable resources (Ghasemaghaei, 2021). Many studies have deemed “firm capability” as a mediator between resources and firm performance (Belhadi et al., 2021; Lou and Wu, 2021; Mikalef and Gupta, 2021). Firm capabilities are vital attributes required for business operations (Yao et al., 2021). These capabilities help deploy other necessary resources to improve firm performance (Yao et al., 2021). We focus on the firm capability in creating value because AIC can enhance the firm’s capabilities and improve firm performance (Chatterjee et al., 2021a). RBV is frequently used to demonstrate the association among firm resources, capabilities, and performance (Barney, 1991; Chen and Lin, 2021; Hossain et al., 2021; Rahman et al., 2021). Therefore, this study will utilize the RBV in the following processes.

### Artificial Intelligence Capability

**Figure 1:**
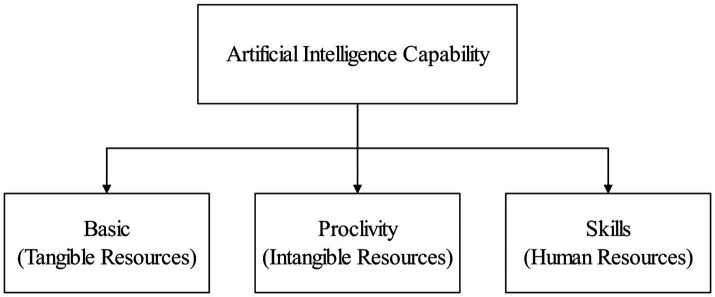
Constructs of artificial intelligence capability (AIC) in e-commerce firms.

Artificial intelligence has gained much attention as a potential to increase the competitive advantage of firms (Hughes et al., 2020; Obschonka and Audretsch, 2020; Shareef et al., 2021). Firm capabilities of applied AI also appear to be particularly valuable (Yu et al., 2021). Combined with RBV, we define AIC of e-commerce firms as the ability of firms to build, integrate, and utilize AI-based related resources (Łapińska et al., 2021; Mikalef and Gupta, 2021). Effective and efficient implementation of AI in firms, on the other hand, requires significant infrastructural resources (tangible resources; Bag et al., 2021c; Chatterjee et al., 2021a), which includes financial support (Łapińska et al., 2021), data (Herhausen et al., 2020; Hwang and Kim, 2021), hardware devices and software (Zhang et al., 2020), and technical support (Rahman et al., 2021). The majority of the businesses across E-commerce companies are throughout the Internet, and thus they have a natural advantage in acquiring data resources (Wang and Fan, 2021). While the firm sets down the basic tangible resources, it is significant to efficiently employ the proclivity of using AI (intangible resources; Ashaari et al., 2021; Yu et al., 2021). If firms are not inclined to implement AI in planning, coordination, control, and implementation (Belhadi et al., 2021; Chen and Lin, 2021), even if they have access to very superior AI base resources will not help (Denicolai et al., 2021). With tangible resources (base resources) and intangible resources (proclivity), technical skills (human resources) should also be taken into account (Baldegger et al., 2020; Bag et al., 2021c). Employees’ skills expertise would restrict the difficulty of spreading AI-related technologies (Chatterjee et al., 2021a; Chen and Chen, 2021). Understanding the scope of AI applications and acquiring skills and expertise in using AI systems are prerequisites for employees to apply AI shortly (Vrontis et al., 2021). Therefore, this study constructs three types of resources (shown in Figure 1) for AIC in e-commerce firms: basic (tangible resources), proclivity (intangible resources), and skills (human resources).

### Hypothesis Development

Firm performance (FP) is an important indicator to assess the business’s financial, operational, marketing, and team cooperation (Dubey et al., 2020). Artificial intelligence positively impacts company health and business performance (Yasmin et al., 2020). AIDDM enables firms to systematically collect, evaluate, and analyze the analytics recommended by artificial intelligence systems enhancing decision-making quality and efficiency (Ashaari et al., 2021). Artificial intelligence can collect and comprehend realistic solutions to complex problems (Awan et al., 2021), providing a more reliable decision-making basis (Elia et al., 2021). Artificial intelligence systems provide business managers information transformed from data so that administrators and business executives can solve existing and potential problems (Ashaari et al., 2021). Corporate executives gradually pay more attention to AIDDM as the evidence reveals it can facilitate business innovation (Chaudhuri et al., 2021), supply chain resilience (Zhang et al., 2021), efficiency gains, cost savings, product quality improvements, and customer service improvements (Bag et al., 2021c). AI-assisted decision-making based on artificial intelligence significantly benefits organizations to improve business operational efficiency and performance (Ashaari et al., 2021). Firms that use AIDDM can achieve higher productivity and superior performance (Chatterjee et al., 2021a). Based on the analysis above, the proposed hypotheses are:

*Hypothesis 1 (H1)*: A positive impact of AIDDM on FP.

Firm creativity (FC) is an essential driver of innovation and competitive advantage for firms (Kim et al., 2021a). This study defines firm creativity as the ability of a firm to create novel and valuable ideas (Ferreira et al., 2020). Today’s business environment has become increasingly complex and fluid, and creative organizations tend to be more likely to experiment with new technologies and incorporate them into their daily operations (Liu et al., 2020b). Firms that appreciate creativity are also more willing to attempt the latest technologies, such as artificial intelligence, big data, and cloud computing to convert business processes and decision-making mechanisms (Mikalef and Gupta, 2021). Firm creativity is a necessary antecedent that influences firms to compose strategic thinking and decisions (Dixit et al., 2021). Artificial intelligence management (AIM) refers to a firm support management system assisting the implementation of artificial intelligence (Bag et al., 2021a; Łapińska et al., 2021). Such initiatives depend heavily on the commitment of firms to implement AI technologies (Haesevoets et al., 2021). AIC is firm competitiveness requiring multiple resources to complement each other but can only be guaranteed through long-term monitoring activities (Raisch and Krakowski, 2021). The system development and its updating for artificial intelligence management can improve firms’ quality decisions (Saenz et al., 2020), thus driving better returns for firms (Blohm et al., 2020). The nature of artificial intelligence is a decision-making technique associated with artificial intelligence techniques (Verganti et al., 2020). Suggestions from artificial intelligence consulting systems can affect firms’ decisions (Keding and Meissner, 2021), and firms that master AIC may be willing to choose AI to navigate their decisions (Ashaari et al., 2021). We therefore propose:

*Hypothesis 2 (H2)*: There is a positive impact of FC on AIDDM.

*Hypothesis 3 (H3)*: There is a positive impact of AIM on AIDDM.

*Hypothesis 4 (H4)*: There is a positive impact of AIC on AIDDM.

Artificial intelligence offers creative statements and solutions for firms (Paschen et al., 2020), positively affecting firms’ creativity enhancement (Amabile, 2020). The AIC takes many repetitive tasks and supplies more solutions for firms encountering complex issues (Raisch and Krakowski, 2021). With AIC, firms can devote more human resources to creative activities (Mikalef and Gupta, 2021). The value of AI is closely related to AIM systems (Bag et al., 2021a). Companies can also develop AIC while enhancing the formation and use of AIM systems (Metawa et al., 2021). The implementation of artificial intelligence management systems needs to be driven by various resources of AIC (Rahman et al., 2021). Firms can automate management activities with the help of AI technologies, and firms with AIC can advance AIM (Bag et al., 2020a). In summary, we hypothesize that:

*Hypothesis 5 (H5)*: There is a positive impact of AIC on FC.

*Hypothesis 6 (H6)*: There is a positive impact of AIC on AIM.

In this scenario, IC is an enterprise culture that encourages innovation (Khattak et al., 2021), encouraging motivation and adopting technologies like artificial intelligence within firms (Zhang et al., 2021), and it helps firms reach higher business goals (Chaudhuri et al., 2021). In the face of intense market competition, encouragement of firm innovation is more likely to transform processes and decisions with digital technologies represented by artificial intelligence to gain new business opportunities and improve performance (Yu et al., 2021). Innovation culture plays an essential role in organizations adopting new decision-making approaches and transforming organizations (Chen and Lin, 2021), and AI-based driven decision-making implies opportunities to enhance business performance (Ashaari et al., 2021). Therefore, the hypothesis proposed in this study is:

*Hypothesis 7 (H7)*: There is a positive moderating effect of IC on the relationship between AIDDM and FC.

*Hypothesis 8 (H8)*: There is a positive moderating effect of IC on the relationship between FC and AIDDM.

This study defines ED as modifications and uncertainties in a firm’s external business environment (Dubey et al., 2020; Haftor et al., 2021). Though external elements mainly influence ED, it affects internal management and decision-making (Belhadi et al., 2021). Firms also respond to changes in the external environment with appropriate strategies under management and decision-making (Haftor et al., 2021). Unforeseen circumstances may cause instability in customer demand and uncertainty in product supply, which may require more flexible management strategies to cope with it (de Haas et al., 2020; Sheth, 2020). Firm performance relies on the external environment because the firm cannot run the business independently without supply and demand (Dubey et al., 2020). The moderating effect of environmental dynamism affects firm performance with its antecedent variables (Wamba et al., 2020). Moderate environmental dynamism can also positively affect AIDDM and firm performance (Dubey et al., 2020). In short, we propose the hypothesis:

*Hypothesis 9 (H9)*: There is a positive moderator of ED on the relationship between AIDDM and FP.

*Hypothesis 10 (H10)*: There is a positive moderation of ED on the relationship between AIM and AIDDM.

Firm performance may vary depending on the different firm features (Zhang et al., 2020), such as the age (Bag et al., 2021b; Chen and Chen, 2021) and the number of employees (Pinheiro et al., 2021; Arias-Pérez and Vélez-Jaramillo, 2022). We construct these two features as control variables in this study.

### Conceptual Model

**Figure 2:**
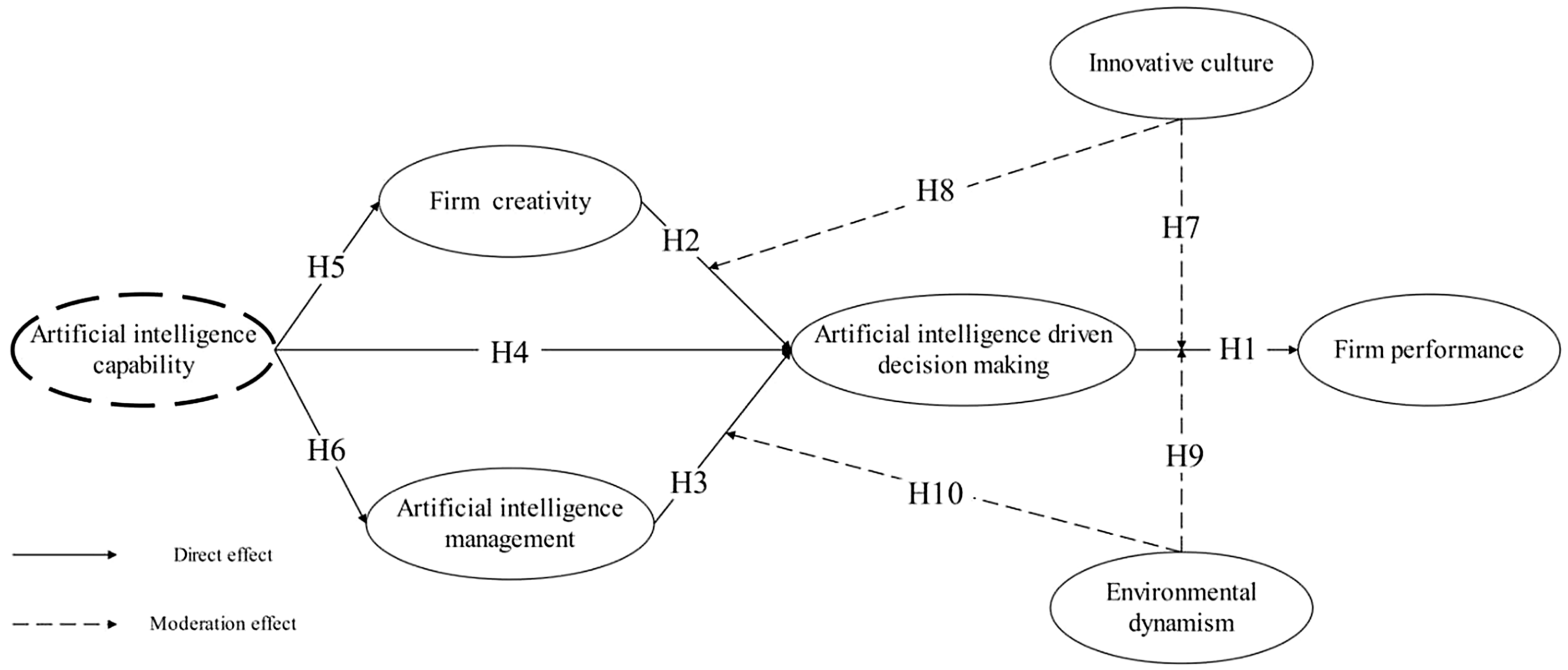
Conceptual model.

This study aims to explore the impact of AIC on e-commerce firm performance. We incorporated the independent variables (AI capability), moderating variables (innovation culture and environmental dynamism), mediating variables (firm creativity, AIM, and driving decision making), and dependent variables (firm performance) based on the research results related to RBV, AI and firm performance. The proposed research model is shown in Figure 2.

## Materials and Methods

### Measurement Scale

To achieve the objectives of this study, scales were developed for data collection (Ghasemaghaei, 2021; Kar et al., 2021). The scale used in this study was adapted under the extant literature (Payne et al., 2021; Yao et al., 2021), and the scale development was divided into three phases. We developed the initial phase’s initial scale in the first phase through a theoretical and literature review.

In the second phase, an English questionnaire was prepared and then translated into Chinese, with the Chinese version subsequently back-translated by a third party to ensure accuracy (Zhang et al., 2020; Wang and Fan, 2021). We sent the draft to six researchers and practitioners to acquire feedback on each questionnaire item to ensure that language did not restrict the understanding of the scales. Several consultations and revisions developed the measurement scales for the pilot survey (Akram et al., 2018a).

In the third phase of developing the questionnaire, we conducted a pilot survey of 30 executives in the Chinese e-commerce industry. We modified the wording of the instrument based on the feedback received. Table 1 presents each construct’s measurement questions and references to support the theoretical framework. The instrument was designed and developed based on a five-point Likert scale (1 = “strongly disagree”; 2 = “disagree”; 3 = “neutral”; 4 = “Agree”; 5 = “Strongly Agree”), which has been widely used by researchers in business management research in the past (Chatterjee et al., 2021a; Chaudhuri et al., 2021; Chen and Chen, 2021; Denicolai et al., 2021; Mostafiz et al., 2021; Rahman et al., 2021).

**Table 1:** Measurement scale.

Code	Items
Artificial intelligence management (AIM) adapted from Bag et al. (2021a).	
AIM1	We employ an artificial intelligence system.
AIM2	We continuously monitor the progress of the AI system.
AIM3	We continuously update the AI system.
Firm performance (FP) adapted from Chatterjee et al. (2021a,b).	
FP1	We are growing our market share faster.
FP2	We are not currently experiencing financial difficulties.
FP3	We continue to introduce new products and services.
FP4	AI implementation is helping to improve business performance.
Firm creativity (FC) adapted from Mikalef and Gupta (2021).	
EC1	We generate many new and useful ideas.
EC2	Our firm climate helps generate new and useful ideas.
EC3	We believe it is important to generate new and useful ideas.
Artificial intelligence driven decision making (AIDDM) adapted from Ashaari et al. (2021).	
AIDDM1	We believe it is important to have, understand and use AI.
AIDDM2	We rely on AI to support decision-making.
AIDDM3	We develop new strategies based on AI.
AIDDM4	We need AI for effective decision-making.
Environmental dynamism (ED) adapted from Dubey et al. (2020, 2021).	
ED1	We can change the efficiency of our operations in response to demand.
ED2	Our marketing strategy is changing rapidly during the crisis.
ED3	The supply and demand side is very unpredictable during a crisis.
ED4	We are adopting artificial intelligence technologies to improve entrepreneurial performance in response to the crisis.
Artificial intelligence basic (AIB) adapted from Mikalef and Gupta (2021) and Belhadi et al. (2021).	
AIR1	We have the hardware equipment (computers, etc.) to apply AI.
AIR2	We have the technical resources to apply AI.
AIR3	We have the software to apply AI (AI software, etc.).
AIR4	We have access to the data needed to run AI.
AIR5	We have arranged sufficient funding for AI projects.
Artificial intelligence skills (AIS) adapted from Zhang et al. (2020) and Mikalef and Gupta (2021).	
AIS1	We understand the range of applications of AI.
AIS2	We can develop plans for the use of AI.
AIS3	We have the skills to apply AI.
AIS4	We have access to training in the use of AI.
AIS5	We can use AI technologies.
Artificial intelligence proclivity (AIP) adapted from Belhadi et al. (2021) and Mikalef and Gupta (2021).	
AIP1	We have a recognition of the importance of innovation.
AIP2	We have a strategy for developing innovation efforts.
AIP3	We can implement innovation programs.
AIP4	We will introduce new products or technologies to improve business performance.
AIP5	We will take aggressive action to capitalize on growth opportunities.
Innovative culture (IC) adapted from Khattak et al. (2021).	
IC1	Our flexible organizational structure helps integrate different perspectives.
IC2	We take risks by constantly trying new ways of doing things.
IC3	Our culture encourages innovation.

### The Second-Order Formative Construct of AIC

When a latent variable is not directly associated with a measured variable, but is initially associated with a lower-order latent variable, and the lower-order latent variable is then associated with a measured variable, depending on the level of association, second-order variables, third-order variables, etc. can be formed (Hair et al., 2022). The association between variables can be reflective or formative (Becker et al., 2012). For second-order variables, four types exist: reflective-reflective, reflective-formative, formative-reflective, and formative-formative (Sarstedt et al., 2019). Artificial intelligence requires the application of many complementary resources to enhance business performance (Ghasemaghaei, 2021; Mikalef et al., 2021). Combining the current research outcomes, we constructed the AIC as a formative second-order latent variable consisting of three first-order variables (Table 2): basic, proclivity, and skills (Ashaari et al., 2021; Chatterjee et al., 2021a; Mikalef and Gupta, 2021).

**Table 2:** The formative construct of AIC.

Second-order	Type	First-order	Type
Artificial intelligence capability	Formative	Basic (Tangible Resources)	Formative
		Proclivity (Intangible Resources)	Formative
		Skills (Human Resources)	Formative

### Data Collection

We operated a cross-sectional survey to experiment with the theoretical model (Akram et al., 2020, 2021; Chen and Siau, 2020; Dubey et al., 2020; Haftor et al., 2021; Łapińska et al., 2021), and the online questionnaire was designed utilizing WJX.cn (an online questionnaire tool; Payne et al., 2021; Yao et al., 2021). This study conducted a targeted target audience survey through e-commerce associations, WeChat communities of e-commerce entrepreneurs (Chaudhuri et al., 2021). We received 441 responses within a 4-week data collection period. Respondents were informed of the purpose of the survey, data usage, and information protection at the time of invitation, and they also had the right to withdraw from the survey at any time (Bag et al., 2020a; Chatterjee et al., 2021b). We set up not to allow submission of questionnaires with omissions; thus, there were no incomplete questionnaires in this survey (Chen and Siau, 2020; Bag et al., 2021b). After data cleaning (Bag et al., 2021c; Chatterjee et al., 2021a; Zhang et al., 2021), we obtained 394 valid questionnaires with a validity rate of 89.3%. Table 3 presents the characteristics of the interviewed organizations.

**Table 3:** Characteristics of the sample.

Characteristics	Number (*n* = 394)	%
**Firm age**		
<1	67	17.0
1–3	142	36.0
4–6	131	33.2
>6	54	13.7
**No. of employees**		
1–5	110	27.9
5–10	141	35.8
>10	143	36.3
**Industry type** Online retail outlets	394	100

### Data Analysis

Structural equation modeling (SEM) has the advantage of examining the interrelationships among multiple independent variables and one or more dependent variables (Belhadi et al., 2021; Łapińska et al., 2021). As an exploratory study, the most appropriate approach for this paper would be the PLS-SEM (Chatterjee et al., 2021b). The proposed research model contains higher-order variables (Ashaari et al., 2021) testing by the PLS-SEM (Bag et al., 2021a). In addition, PLS-SEM techniques in business performance (Chatterjee et al., 2021a; Chaudhuri et al., 2021; Pinheiro et al., 2021; Shao et al., 2021) and artificial intelligence research (Khalid, 2020; Bag et al., 2021b; Mikalef and Gupta, 2021; Rana et al., 2021) have been involved for a long time. Ultimately, we determined to use the PLS-SEM analysis in Smart PLS 3 software to test the hypotheses and theoretical models (Chen and Siau, 2020; Hair et al., 2022).

### Common Method Bias

This study endeavors to reduce the impact of common method bias (CMB). At the time of the questionnaire, it was communicated that the study was intended for academic use and that the entire process would not involve respondents’ private information (Chatterjee et al., 2021b). After completion of the survey, we conducted Harman’s one-factor test with the help of SPSS 25 (Bag et al., 2020a; Nasiri et al., 2021), and comparing variance inflation factor (VIF) values (Bag et al., 2021b) to test for the presence of CMB. The results of Harman’s one-factor test indicated that the first factor explained 29.9% of the variance (<50%), and it can be inferred that CMB does not have a serious impact on the study results (Nasiri et al., 2021). Furthermore, the VIF of potential variables in the study model was below a threshold value of 5. These findings support that the CMB does not seriously impact the study results (Haftor et al., 2021; Hair et al., 2022).

### Non-response Bias

Since we used a questionnaire for data collection, it was essential to test non-response bias (NRB; Chaudhuri et al., 2021). The presence of NRB was examined by comparing the data collected from the survey (top 25% of respondents and last 25% of respondents; Rahman et al., 2021). Our *t*-test results between early and late respondents using SPSS 25 showed no statistically significant difference between these two groups (*p* > 0.05; Bag et al., 2021a). Thus, we believe that NRB will not affect the results of our further analysis (Yao et al., 2021).

## Results

Partial least squares structural equation modeling assessment involves two key components: measurement model assessment and structural model assessment (Haftor et al., 2021; Hair et al., 2022). Measurement models examine convergent and discriminant validity, while structural models investigate the relationship among constructs (Ashaari et al., 2021). We used SmartPLS 3 software to analyze our data (Pinheiro et al., 2021).

### Measurement Model

Table 4 shows the reliability and validity of the constructs. It shows that the Cronbach alpha is higher than 0.7 for all the constructs, the composite reliability (CR) is also higher than 0.7, the Rho_A values are not less than 0.7, and the factor loadings are higher than 0.7 for all the items. In addition, the average variance extracted (AVE) was also above 0.5, which confirms the convergent validity (Wang et al., 2021; Hair et al., 2022). The scales developed in this study were adapted from existing literature. The underwent multiple rounds of revision and pre-research, resulting in good content validity of the model in this study (Wang et al., 2021; Hair et al., 2022). Discriminant validity was determined using AVE square root, heterotrait-monotrait ratio (HTMT), and cross-loading (Wang et al., 2021; Hair et al., 2022). We found higher correlations between potential constructs than the square root of AVE for each construct. The loadings for each metric were higher than the respective cross-loadings, indicating sufficient discriminant validity of the measurement model (Table 5). The HTMT values for all constructs were less than the critical value of 0.9 (Table 6). Therefore, we determined that the measurement model had sufficient discriminant validity (Wang et al., 2021; Hair et al., 2022). In conclusion, we confirmed that the measurement model has sufficient reliability and validity for the next structural model analysis step.

**Table 4:** Reliability, convergent validity, and discriminant validity.

Construct	AIP	AIB	AIS	AIDDM	AIM	ED	FC	FP	IC
AIP	n/a								
AIB	0.676	n/a							
AIS	0.693	0.656	n/a						
AIDDM	0.341	0.380	0.422	0.826					
AIM	0.453	0.451	0.466	0.522	0.866				
ED	0.204	0.115	0.144	0.293	0.218	0.843			
FC	0.415	0.426	0.489	0.555	0.332	0.104	0.870		
FP	0.344	0.357	0.387	0.650	0.421	0.249	0.532	0.842	
IC	0.083	0.114	0.153	0.332	0.175	0.166	0.241	0.386	0.856
Cronbach’s Alpha	n/a	n/a	n/a	0.845	0.833	0.865	0.839	0.863	0.819
Rho_A	n/a	n/a	n/a	0.846	0.834	0.869	0.844	0.865	0.822
CR	n/a	n/a	n/a	0.896	0.900	0.908	0.903	0.907	0.892
AVE	n/a	n/a	n/a	0.683	0.749	0.711	0.757	0.710	0.733

Square root of average variance extracted (AVE) in diagonals.

**Table 5:** Factor loadings and cross loadings.

Items	AIDDM	AIM	AIP	AIB	AIS	ED	FC	FP	IC
AIDDM1	0.802	0.379	0.273	0.311	0.351	0.205	0.431	0.521	0.296
AIDDM2	0.839	0.482	0.283	0.324	0.359	0.271	0.501	0.557	0.287
AIDDM3	0.842	0.412	0.271	0.322	0.362	0.254	0.446	0.522	0.257
AIDDM4	0.820	0.445	0.300	0.297	0.323	0.234	0.453	0.546	0.257
AIM1	0.453	0.873	0.384	0.403	0.408	0.156	0.280	0.368	0.147
AIM2	0.466	0.863	0.419	0.385	0.407	0.211	0.289	0.368	0.165
AIM3	0.434	0.861	0.372	0.382	0.396	0.198	0.294	0.358	0.141
AIP1	0.269	0.362	0.806	0.563	0.569	0.152	0.344	0.276	0.069
AIP2	0.313	0.343	0.755	0.523	0.522	0.140	0.364	0.287	0.071
AIP3	0.242	0.319	0.799	0.542	0.521	0.171	0.306	0.245	0.067
AIP4	0.252	0.353	0.820	0.507	0.577	0.149	0.296	0.266	0.073
AIP5	0.291	0.432	0.820	0.569	0.583	0.203	0.353	0.300	0.050
AIB1	0.248	0.386	0.548	0.805	0.545	0.112	0.305	0.211	0.065
AIB2	0.327	0.357	0.548	0.865	0.552	0.099	0.338	0.296	0.102
AIB3	0.320	0.328	0.575	0.791	0.505	0.102	0.364	0.331	0.085
AIB4	0.313	0.339	0.506	0.780	0.509	0.026	0.367	0.315	0.153
AIB5	0.335	0.418	0.565	0.817	0.550	0.125	0.357	0.297	0.059
AIS1	0.356	0.326	0.539	0.547	0.826	0.154	0.396	0.312	0.124
AIS2	0.370	0.387	0.601	0.557	0.829	0.121	0.403	0.344	0.123
AIS3	0.356	0.392	0.585	0.551	0.816	0.097	0.416	0.314	0.086
AIS4	0.311	0.374	0.556	0.502	0.832	0.128	0.367	0.298	0.150
AIS5	0.355	0.449	0.589	0.560	0.842	0.098	0.441	0.335	0.152
ED1	0.261	0.187	0.207	0.098	0.144	0.870	0.075	0.240	0.100
ED2	0.254	0.224	0.131	0.108	0.129	0.843	0.093	0.215	0.172
ED3	0.247	0.155	0.193	0.111	0.139	0.822	0.116	0.194	0.114
ED4	0.222	0.163	0.154	0.069	0.066	0.838	0.067	0.187	0.181
FC1	0.433	0.276	0.350	0.362	0.408	0.091	0.857	0.445	0.185
FC2	0.490	0.294	0.415	0.406	0.488	0.072	0.907	0.462	0.207
FC3	0.522	0.296	0.316	0.341	0.375	0.110	0.845	0.483	0.236
FP1	0.519	0.351	0.296	0.313	0.326	0.206	0.438	0.823	0.329
FP2	0.561	0.353	0.286	0.286	0.316	0.195	0.462	0.878	0.315
FP3	0.560	0.336	0.254	0.297	0.310	0.201	0.438	0.863	0.358
FP4	0.548	0.381	0.323	0.308	0.354	0.240	0.454	0.802	0.299
IC1	0.275	0.127	0.068	0.078	0.128	0.099	0.207	0.307	0.850
IC2	0.282	0.148	0.067	0.132	0.120	0.131	0.177	0.317	0.850
IC3	0.295	0.172	0.076	0.083	0.144	0.190	0.232	0.365	0.869

**Table 6:** Assessment of discriminant validity using heterotrait-monotrait ratio (HTMT).

Construct	AIDDM	AIM	ED	FC	FP	IC
AIDDM						
AIM	0.619					
ED	0.340	0.255				
FC	0.657	0.397	0.123			
FP	0.760	0.497	0.288	0.626		
IC	0.399	0.210	0.197	0.289	0.458	

### Formative Constructs Validation

**Figure 3:**
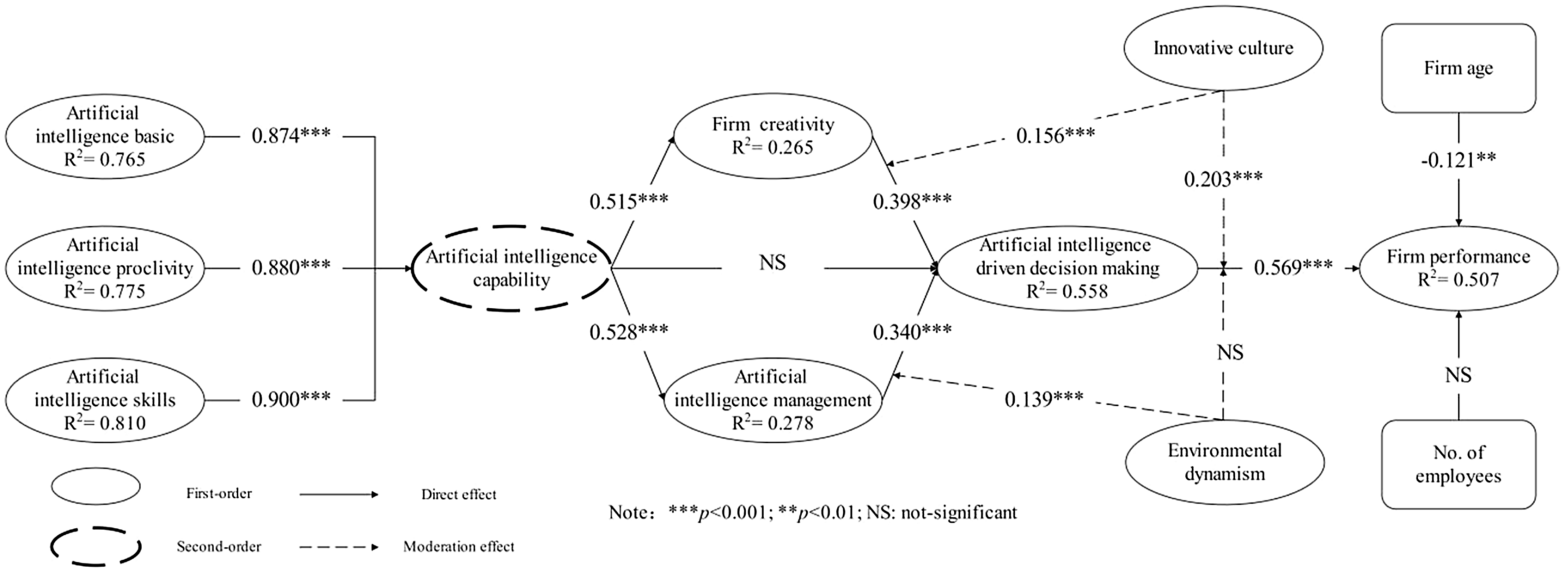
Partial least squares structural equation modeling (PLS-SEM) results.

As suggested by Hair et al. (2022), we tested the formative structure of AI ability with the help of SmartPLS 3. Table 5 displays that the significance among AIC and all three first-order constructs is less than 0.001, indicating that AIC is well-constructed second-order models (Figure 3; Becker et al., 2012; Hair et al., 2022). The results suggest that AIC are higher-order models constructed from three first-order constructs: basic, proclivity, and skills (Table 7).

**Table 7:** Formative constructs validation.

Constructs	Measures	Weighting	*t* values	Significance	VIF
Basic	AIB1	0.240	3.143	*p < 0.01*	2.113
	AIB2	0.106	1.301	*NS*	2.119
	AIB3	0.294	4.487	*p < 0.001*	2.026
	AIB4	0.239	3.279	*p < 0.01*	2.166
	AIB5	0.355	4.975	*p < 0.001*	2.221
Skills	AIS1	0.165	2.334	*p < 0.05*	2.113
	AIS2	0.310	4.853	*p < 0.001*	2.119
	AIS3	0.310	4.848	*p < 0.001*	2.026
	AIS4	0.088	1.242	*NS*	2.166
	AIS5	0.323	4.362	*p < 0.001*	2.221
Proclivity	AIP1	0.268	4.05	*p < 0.001*	1.885
	AIP2	0.304	4.857	*p < 0.001*	1.631
	AIP3	0.168	2.679	*p < 0.01*	1.857
	AIP4	0.136	1.866	*NS*	2.000
	AIP5	0.368	5.318	*p < 0.001*	1.999
Constructs	Measures	**Path coefficient**	*t* values	Significance	VIF
Artificial intelligence capability	Basic	0.874	58.292	*p < 0.001*	1.000
	Skills	0.900	71.305	*p < 0.001*	1.000
	Proclivity	0.880	62.389	*p < 0.001*	1.000

### Structural Model

After examining the measurement model’s reliability, validity, and formative structure, this study will analyze the data to examine the relationship among the variables (Ashaari et al., 2021; Hair et al., 2022). Figure 3 shows the results after bootstrapping. We found a positive effect of AIDDM on FP (*β* = 0.569; *p* < 0.001), indicating that AIDDM increased firm performance. We also found a positive effect of both FC (*β* = 0.398; *p* < 0.001) and AIM (*β* = 0.340; *p* < 0.001) on AIDDM. There was a positive effect of AIC on both FC (*β* = 0.515; *p* < 0.001) and AIM (*β* = 0.528; *p* < 0.001). For the control variables, firm age negative affects firm performance (*β* = −0.121; *p* < 0.01) and number of employees does not affect firm performance (*β* = 0.016; *p* > 0.05). Therefore, hypotheses H1, H2, H3, H5, H6, H7, H8, and H10 are supported, and hypotheses H4 and H9 are not supported. The *R*^2^ value of firm performance in the model is 0.507, and this result indicates the good explanatory power of the model (Hair et al., 2022).

The *R*^2^ value of firm performance in the model is 0.507, which suggests the model’s good explanatory power (Hair et al., 2022). The predictive relevance *Q*^2^ value in the model is 0.347 (>0), which verifies that the model has appropriate predictive relevance (Hair et al., 2022). The result of standardized root mean square residual (SRMR), an indicator of model fitness, was 0.079 (<0.08), implying that the model proposed in this study has a good fitness (Hair et al., 2022).

### Moderating and Mediating Effect

We used bootstrapping in SmartPLS to examine the mediating and moderating effects (Akram et al., 2018b; Hair et al., 2022). The test results for mediating effects (Table 8) exhibited a mediating effect of FC and AIM on the relationship between AIC and AIDDM. Both AIM and FC indirectly affected FP through AIDDM. Four moderating effects were examined in this study (Figure 3 and Table 8). There was a positive moderating effect of IC on the relationship between AIDDM and FP (*β* = 0.203; *p* < 0.001), and IC also had a positive moderating effect on the relationship between FC and AIDDM (*β* = 0.156; *p* < 0.001). ED showed a positive moderating effect on the relationship between AIM and AIDDM (*β* = 0.139; *p* < 0.001). However, the moderating effect of ED between AIDDM and FP was insignificant (*β* = 0.016; *p* > 0.05).

**Table 8:** Results of meditation and moderation.

Effect	Relationships	Path coefficient	STDEV	*t* values	Results
Meditation	AIC → AIM → AIDDM	0.180	0.026	6.872^***^	Supported
	AIC → FC → AIDDM	0.205	0.026	7.915^***^	Supported
	AIC → AIDDM → FP	0.015	0.025	0.591	Not supported
	AIM → AIDDM → FP	0.194	0.027	7.25^***^	Supported
	FC → AIDDM → FP	0.227	0.030	7.478^***^	Supported
Moderation	ED × AIDDM → FP	0.018	0.038	0.475	Not supported
	ED × AIM → AIDDM	0.139	0.025	5.492^***^	Supported
	IC × AIDDM → FP	0.203	0.042	4.829^***^	Supported
	IC × FC → AIDDM	0.156	0.030	5.127^***^	Supported

*** *p* < 0.001.

## Discussion

### Theoretical Implications

This paper investigates the performance of e-commerce enterprises and proposes a theoretical model incorporating RBV (Barney, 1991; Chatterjee et al., 2021b), which explores the construct of AIC in e-commerce enterprises, finds the effects of firm creativity, AIM, AIDDM on firm performance, and examined the moderating effects of an innovation culture and environmental dynamism. This study reveals the mechanisms that constitute AIC of e-commerce firms and ensures that the AIC of e-commerce firms affects firm performance through creativity, AIM, and AIDDM (Ashaari et al., 2021). This study extends RBV’s research findings on firm creativity, AIM, and AIDDM (Hossain et al., 2021; Rahman et al., 2021). Our research sheds light on the composition mechanism of AIC in e-commerce enterprises and its effect on corporate performance. The data analysis conducted that the AIC of e-commerce firms are second-order variables formed by three first-order variables: basic (tangible resources), proclivity (intangible resources), and skills (human resources; Mikalef and Gupta, 2021). Our proposed AIC for e-commerce firms is a second-order formative model suggesting AIC is constructed by the three complementary resources of basic, proclivity, and skill (Ghasemaghaei, 2021). This classification of the constitutive resources of AIC in e-commerce firms provides additional facilities ensuing investigation and management.

The study outcomes reveal the role of firm creativity, AIM, and driving decision-making in the relationship between AIC and firm performance. AIDDM significantly and positively affects firm performance, similar to conclusions of Ashaari et al. (2021). We found that AIC does not directly affect firm performance (Chen and Lin, 2021; Haftor et al., 2021) but indirectly affects AIDDM and firm performance through firm creativity and AIM. AIC, as firm capabilities requiring numerous resources need to demonstrate their business value through innovative measures and quality decisions (Shi et al., 2020). This study demonstrates the consequence of an innovation culture and environmental dynamism as moderating variables on the relationship between AIC and firm performance. Innovation culture has a positive moderating effect on the relationship between firm creativity and AIDDM (Zhang et al., 2021) and AIDDM and firm performance (Ashaari et al., 2021). Environmental dynamism positively affects the relationship between AIM and AIDDM (Belhadi et al., 2021). However, there is no moderating effect of environmental dynamism on the relationship between AIDDM and firm performance, suggesting difficulties in the external environment’s variability and unpredictability to profit from AIDDM. Thus, we should focus on the positive effects of an innovation culture and environmental dynamism as moderating variables in the research model.

Among the control variables, firm age negatively affects firm performance, and startups are more likely to desire to leverage new technologies and models to improve firm performance (Yao et al., 2021), while more established firms may be a more conservative view of new technologies. The number of firm employees does not affect firm performance, presenting no need to be concerned about the impact of firm employee size when considering the relationship between AI and firm performance.

### Managerial Implications

Enterprises can cultivate firm AIC through three aspects: tangible resources (basic), intangible resources (proclivity), and human resources (skills). This study proves that e-commerce firms AIC is formed by three first-order variables: basic, proclivity, and skills, and the data analysis results indicate that AIC is a well-constructed second-order model. Firms need to make the real business value of AI technology to improve firm performance and cannot rely on either hardware devices or software, technical resources (Rahman et al., 2021), and data resources (Chaudhuri et al., 2021). Nevertheless, these complementary resources should be allowed to construct the superior competitiveness of the firm organically.

Use artificial intelligence to enhance firm creativity. Companies can adopt artificial intelligence technologies to perform repetitive tasks in business operations, release more human resources, and reduce costs (Mikalef and Gupta, 2021). In addition, businesses can also attempt to employ AI for innovative work, using deep mining of internal and external data to discover where the current needs of firm customers are going, thus giving them more time to optimize processes, products, and services. For example, AI technology can integrate solutions that consumers likely favor and record current browsing data, click data and sales data in time to predict the higher quality products and services to meet consumers’ needs.

Foster an internal culture of innovation and keep an eye on external environmental changes. This paper affirms the positive moderating role of an innovation culture and environmental dynamism present in the research model. E-commerce firms should cultivate a culture of innovation that incorporates the employees’ views at all levels within the firm and should also consider the opinions of external experts. Firms can also establish fault-tolerance mechanisms to allow new ideas and solutions, providing more opportunities to improve performance. Changes and unpredictability in the external environment can also affect business operations (Haftor et al., 2021). Firms can use AI technologies to observe changes in the external environment in real-time and recommend intervention strategies to give them insight into business opportunities in a highly competitive market.

Emphasis is on establishing AI to manage and drive decision-making to leverage the positive effects that AI can bring in decision-making. Establishing processes based on AIDDM is an important stage in leveraging AI to enhance business performance (Chatterjee et al., 2021a). Companies can genuinely appreciate the technological dividends of AI by making decisions in considerable areas, such as marketing, product development, and customer relationship management. The rapid development of digital technology also requires firms to establish AIM systems to monitor and update AI systems promptly.

### Limitations and Future Research

This research contributes to current theoretical developments and AI practices, but it solely pays attention to e-commerce firms in China, and we can extend the established theoretical framework to more industries and other countries in the future. The firm performance is derived from the subjective evaluations of the respondents and does not cover the financial data of the sample firms; future studies could consider both qualitative and quantitative methods to explore more correlations and phenomena. This study used cross-sectional data at one point, and it did not consider longitudinal changes in AIC and firm performance. We could focus on other firm characteristics, such as risk-taking, R&D capability, market development capability, and productivity. Future studies will explore more organizational characteristics variables to improve the model presented in this model.

## Conclusion

We clarified and assessed the components of AIC critical for improving firm performance with AI. Therefore, we analyze the components of firms AIC relating to RBV. By reviewing the relevant literature, we proposed a research model of AIC and firm performance in e-commerce businesses, aiming to explain and predict the performance under AI application scenarios. Following a newly developed scale, we designed an online questionnaire and received 394 valid questionnaires. Further data analysis with the SmartPLS 3 utilizes the PLS-SEM analysis technique. The results illustrated that the model proposed in this study has sufficient explanatory power, predictive power, and fitness. We found that AIC as a second-order variable is formed by three first-order variables: basic, skills, and proclivity. AIC indirectly influences firm performance through firm creativity, AIM, and AIDDM. Corporate creativity, AIM, and AIDDM are significant mediating variables between AIC and firm performance. Innovation culture positively moderates the relationship between firm creativity and AIDDM and positively moderates the relationship between AIDDM and firm performance. Environmental dynamism positively moderates the relationship between AIM and AIDDM. Among the control variables, firm age negatively affects firm performance, and the number of firm employees does not affect firm performance. This study’s empirical findings help enterprises to improve firm performance and gain a competitive advantage with the help of AI, enrich the research on AI and firm performance, and contribute to theory and management practice.

## Data Availability Statement

The original contributions presented in the study are included in the article/supplementary material; further inquiries can be directed to the corresponding author.

## Ethics Statement

Ethical review and approval was not required for the study on human participants in accordance with the local legislation and institutional requirements. Written informed consent for participation was not required for this study in accordance with the national legislation and the institutional requirements.

## Author Contributions

DC and SW: conceptualization, validation, and writing—original draft preparation. SW: methodology, software, investigation, and resources. DC, JE, and SW: writing—review and editing. JE: supervision. DC: funding acquisition. All authors contributed to the article and approved the submitted version.

## Funding

This study was funded by Zhejiang Soft Science Project No. 2022C35047; Major Humanities and Social Sciences Research Youth Key Projects in Zhejiang Colleges and Universities under Grant No. 2021QN014; Zhejiang Province Philosophy and Social Science Planning Project under Grant No. 21NDJC172YB; Project of China National Social Science Fund under Grant No. 19BGL084.

## Conflict of Interest

The authors declare that the research was conducted in the absence of any commercial or financial relationships that could be construed as a potential conflict of interest.

## Publisher’s Note

All claims expressed in this article are solely those of the authors and do not necessarily represent those of their affiliated organizations, or those of the publisher, the editors and the reviewers. Any product that may be evaluated in this article, or claim that may be made by its manufacturer, is not guaranteed or endorsed by the publisher.
